# Sex-specific median life expectancies from *ex situ* populations for 330 animal species

**DOI:** 10.1038/sdata.2019.19

**Published:** 2019-02-19

**Authors:** Judy P. Che-Castaldo, Amy Byrne, Kaitlyn Perišin, Lisa J. Faust

**Affiliations:** 1 Alexander Center for Applied Population Biology, Lincoln Park Zoo, Chicago, IL, USA

**Keywords:** Biodiversity, Zoology, Population dynamics

## Abstract

We present life expectancy estimates for hundreds of vertebrate species based on carefully vetted studbook data from North American zoos and aquariums. These data include sex-specific median life expectancies as well as sample size and 95% confidence limits for each estimate. Existing longevity data for animals primarily consist of maximum lifespan values, which are single observations rather than statistically derived estimates of longevity. Moreover, all of our estimates are based on the same type of data and calculated using consistent, standardized methods. To derive these estimates, we conducted Kaplan-Meier survival analyses using individual-level demographic data (*i.e.*, records of birth and mortality events) from studbook records for each *ex situ* population. Our species set represents a range of vertebrate taxa (primarily mammals, birds, amphibians, and reptiles) and diverse life histories. This dataset will have broad utility, not only for informing comparative demographic and life history studies, but also more broadly for any research or conservation application that requires sex- or species-specific life expectancy information.

## Background & Summary

The expected lifespan, age at maturation, clutch size, and interbirth interval are some of the basic life history traits defining the scheduling of events in an organism’s life. The patterns in and covariation among these life history traits have long been of interest to ecologists and evolutionary biologists^1,2^. Researchers have also sought to predict life history patterns based on other biological traits ranging from body size^3^ to hibernation^4^. Descriptive studies of new populations or a group of related taxa often involve a comparison of life history patterns^5,6^. Thus, a wide range of biological research questions require species-specific longevity information to explore.

Existing data for animal longevity^7–9^ typically include values for the maximum observed lifespan of a species. These datasets include a mix of *in situ* and *ex situ* data, with many estimates from unknown (*ex situ* or wild) sources. Maximum lifespans are usually single observations, and therefore can represent outliers rather than biological tendencies. In contrast, we present a dataset that contains rarely available median life expectancy (MLE) values. MLEs provide more robust estimates of species longevity because they are statistically derived from a sample (in our case, typically hundreds) of observed lifespans^10^. Our dataset also differs from existing longevity data because estimates for all species are derived from the same type of data and from the same sources, and calculated using consistent, standardized methods.

Specifically, our longevity estimates are derived from studbooks for animal populations in North American zoos and aquariums. A population consists of all animals of the same species that are cooperatively managed through planned breeding and exchanges. These animals are held in member institutions of the Association of Zoos and Aquariums (AZA) as well as other non-member institutions that collaborate with AZA. The studbooks contain individual-level demographic information (e.g., birthdates, reproduction events, and death dates when applicable) for all animals in the population^11^. These data are carefully vetted by population biologists working with each population, and we then used them to estimate Kaplan-Meier survival curves and to calculate MLE for each species (see Methods).

Our dataset includes over 300 vertebrate species, which primarily consist of mammals and birds but also include a number of reptiles and amphibians (see Data Records). The original motivation for calculating these MLEs was to provide zoo managers with accurate biological information about the animals in their care for communication and outreach purposes. However, these life expectancy estimates have broad utility for any research related to life history theory and comparative demography, and for informing the conservation management of single or groups of species. In addition to the MLE estimates, our dataset also includes the sample size (i.e., the number of individuals whose partial or full lifespans were used in the survival analysis) and 95% confidence limits for each estimate to enhance their utility for research applications.

## Methods

A studbook is an electronic database containing the genetic and demographic information for an *ex situ* population. It includes a record for each (historic and living) individual in the managed population, such as pedigree information and the dates of birth, death, and transfers between institutions (if any). These individual records are compiled by a volunteer studbook keeper who participates in the cooperative management of the population. That is, managers of a population would regularly plan for the breeding and transferring of individuals among institutions, with the goal of maintaining a population that is as genetically diverse and demographically self-sustaining as possible^11^. A trained population biologist typically advises on this planning process by evaluating the population’s genetic and demographic status using studbook data. As part of this process, the biologist validates each studbook through automated error checking and discussions with program leaders to ensure all records are accurate and common errors are corrected. Typical studbook errors affecting longevity estimates include having entries for life events after a death event, missing death events leading to very old individuals, and duplicated entries for an individual. Attempts are made to resolve records for any individuals with unknown birth or death dates.

Based on the validated studbook data, we calculated age-specific survival probabilities using the non-parametric Kaplan Meier survival analysis^12,13^. This method correctly handles both right censoring (individuals that are alive at the end of the analysis window) and left truncation (individuals that enter the dataset after their birthdate), which are common in studbook datasets. We specified timeframes for each population to best reflect modern management (in terms of husbandry, veterinary, and nutrition practices) while maintaining as many individuals in the dataset as possible. The resulting analysis time windows were typically from 1980 or later to the present. The survival probability (*L*) was estimated at every age *t* (measured in days) at which an individual in the sample dies or is censored. For each *t*, the number of deaths observed (*d*_*t*_) and the number at risk (i.e., the number surviving to that age; *N*_*t*_) were tallied. *L*_*t*_ was then calculated as the product of the survival probabilities for each age up to *t* (following Klein and Moeschberger^12^, equation 4.6.1):

$$L_{t}=\prod_{t_{i}\leq t}\left[1-\frac{d_{i}}{N_{i}}\right]$$

By definition, survivorship is 1.0 at the starting age for the analysis, which we set at age 365 days. This was because mortality in the first year of life can be highly dependent both on life history and on the specific management or animal care strategies at the time and may not be generalizable across settings. Estimates of *L*_*t*_ were made until the maximum longevity observed in the dataset.

The exact median life expectancy (or the age *t* for which L_t_ = 0.5) was calculated via linear interpolation, unless one of the Kaplan Meier estimates fell exactly on 0.5. That is, if [x_0_, L_0_] is the smallest [survival time, survival probability] pair greater than L_t_ = 0.5, and [x_1_, L_1_] is the largest pair less than L_t_ = 0.5, then MLE was calculated as (following Klein and Moeschberger^12^, equation 5.4.8):

$$MLE=x_{0}+\left(\frac{\left(L_{0}-0.5\right)\left(x_{1}-x_{0}\right)}{L_{0}-L_{1}}\right)$$

Finally, MLE was divided by 365 to yield units in years rather than days. The number of individuals whose partial or full lifespans were used to calculate the survival curves was recorded as the sample size of the analysis (Data Citation 1).

We calculated 95% log-transformed confidence intervals (CIs) for the MLE estimate by determining the values of L that yielded Z ≤−1.96 and Z ≥ 1.96. Z was calculated for each survival time *t* as (following Klein and Moeschberger^12^; equation 4.5.5):

$$Z=\frac{\left\{\ln\left[-\ln\left(\hat{L}\right)\right]-\ln[-\ln\left(0.5\right)]\right\}[\hat{L}\ln\left(\hat{L}\right)]}{\sqrt{\hat{V}}},$$

where $\hat{L}$ was the survival estimate and $\hat{V}$ is the standard error of $\hat{L}$:

$$\hat{V}={\hat{L}}^{2}\sum_{t_{i}\leq t}\frac{d_{i}}{N_{i}(N_{i}-d_{i})}.$$

For each population, we calculated an overall MLE estimate that is based on the entire studbook dataset, including males, females, and any individuals of unknown sex. We also estimated sex-specific MLEs based on only the male or female data.

### Code availability

We implemented these calculations using the freely available studbook software PopLink version 2.4^14^. An author on this paper (L.J.F.) helped to develop PopLink as a tool to provide standardized analysis and interpretation of studbook data for zoo managers. For our survival analyses, we used the “PopLink Survival Tool,” which runs the calculations as described above and generates an Expert Survival Statistics report for each population. From these reports we compiled the sex-specific MLE estimates, upper and lower 95% confidence limits, and sample sizes for each population into the current dataset. Because Survival Statistics reports are generated whenever a population undergoes breeding and transfer planning, new estimates are continually produced. An up-to-date set of MLE estimates will be maintained on the AZA website (https://www.aza.org/species-survival-statistics), however this table does not contain the confidence limits or sample sizes associated with the estimates.

## Data Records

The full MLE dataset has been deposited in figshare (Data Citation 1). The table included the species’ common name, scientific name, taxonomic group, sample size, MLE estimates (in years), and the lower and upper 95% confidence limits (in years). The last four variables were separated into sex-specific columns: one set for the overall MLE estimates (those calculated from all studbook data including unknown-sex individuals), one set for male MLE estimates (those calculated from male data only), and one set for female MLE estimates (those calculated from female data only). Two additional columns in the dataset indicated whether the male or female MLE estimates were based on datasets that failed at least one of the five data quality tests (Data Deficient = “yes”; see Technical Validation).

**Figure 1:**
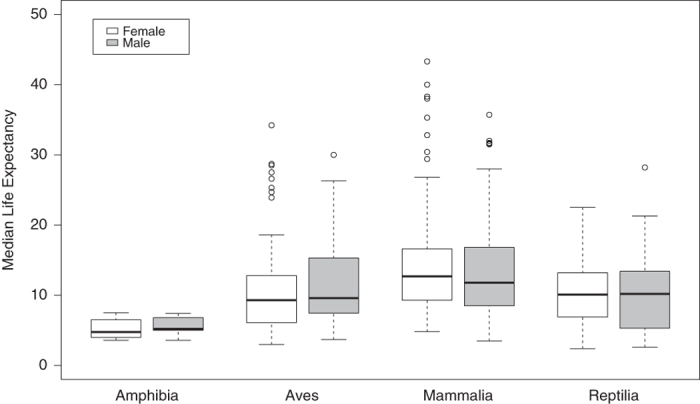
Modified boxplot of median life expectancy estimates (MLE; in years) by sex and taxonomic class, based on *ex situ* populations for 327 species. Data for two Chondrichthyes and one Arachnida species are not shown. Thick lines represent medians; box edges represent the 25^th^ and 75^th^ quartiles; lower edges of whiskers represent the 25^th^ quartile minus 1.5 times the interquartile range or the minimum MLE value, whichever is greater; and upper edges of whiskers represent the 75^th^ quartile plus 1.5 times the interquartile range or the maximum MLE value, whichever is smaller; open circles represent MLE values that fall outside the range of the whiskers.

In total, our dataset included entries for 330 unique species or subspecies. Some populations had sufficient demographic data to reliably estimate MLE only for the overall population or only for one sex. Excluding the estimates based on insufficient studbook data, our dataset contained estimates of female MLE for 270 populations and of male MLE for 258 populations. Table 1 presents summary statistics for the MLE estimates, and the distribution of MLEs across species by sex and taxonomic group are shown in Fig. 1. The figure does not include MLE estimates for two fish populations (ocellated river stingray, sand tiger shark) and one invertebrate population (Mexican red-kneed tarantula) that are also included in our dataset.

The studbook data used to calculate MLEs represented thousands of animals in AZA member institutions and other collaborating institutions, the majority of which are located in the United States. Most of the MLEs came from survival analyses that were conducted in the past 5 years (309 of 330 records were from 2014–2018), and the remaining were analyzed from 2010–2013. In terms of sample sizes, the smallest analysis that still produced a sex-specific MLE estimate included 41 individuals (for female Madagascar buttonquail), whereas the largest included 1425 individuals (for male Caribbean flamingo). The mean sample size across species was 254.1 (±209.4 SD) individuals for female MLEs, and 242.1 (±198.7 SD) individuals for male MLEs.

## Technical Validation

We ensured the technical quality of our life expectancy dataset by carrying out validation procedures at three points in our calculations. First, each studbook was vetted by the population biologist who was advising the *ex situ* population (see Methods). Second, studbook records with high uncertainty were excluded from the survival analysis. Specifically, individuals with unknown birthdates, unknown dates of other important events (e.g., transfers between institutions, death), and/or events at unknown or unrecognized institutions were not included in the calculations. Third, the PopLink program applied five data quality tests to determine whether each studbook contained sufficient data to provide reliable estimates of MLE:

1. Can the MLE be calculated? For example, median life expectancy cannot be calculated if no deaths were observed in the studbook.
2. Is the sample size (number of individuals at risk) in the first age class of the analysis greater than 30 individuals? The threshold of 30 was chosen based on previous recommendations for life table calculations^11^.
3. Is the sample size (number of individuals at risk) greater than 20 individuals at the median? The lower threshold of 20 was chosen based on discussions with experts on *ex situ* population demography during development of the PopLink software, as a compromise between robustness of the estimates and data availability (due to the smaller sizes of many *ex situ* populations).
4. Are both the upper and lower 95% confidence limits for the MLE estimate defined?
5. Is the MLE estimate sufficiently precise? This test is failed when the length of the 95% confidence interval is greater than 33% of the maximum longevity.

If the studbook dataset failed any of these tests, MLE cannot be reliably estimated and the estimate is marked as being “Data deficient,” although a value may still be reported. We included these values in our dataset as it may still be informative to know that we have attempted to evaluate life expectancy for those species. However, we recommend those values be excluded from formal analysis for the most reliable results.

## Usage Notes

Our dataset has broad utility for a wide range of biological studies including comparative demography and testing theories on life history evolution. For example, analysis of these data can help to delineate patterns of ageing across taxa and to predict other species traits based on lifespan. The MLE estimates may also be used in conservation applications to inform species management, as longevity will affect the scope and duration of the management actions that may be needed. Because lifespan likely differs between *ex situ* and *in situ* settings, our dataset may only provide approximations of the expected longevity for wild animals. Unfortunately, existing large-scale datasets on animal lifespans from wild populations^7–9^ mostly consist of maximum longevity values, and therefore are not comparable with our median life expectancy estimates. Of course, researchers with demographic data from wild populations to estimate life expectancies will be able to compare against our dataset to assess differences between *in situ* and *ex situ* longevity. The most powerful use of our dataset will lie in comparative analyses, because our MLE estimates are derived using the same data sources and consistent methodology for all species, providing reliable quantitative data on the relative longevity among a diverse set of species.

For accurate interpretation of our dataset, we reiterate that the starting age for our survival analysis was set at 365 days for all species. Individuals in the studbook that did not survive to their first birthday were excluded from the survival analyses. Users should interpret our MLE estimates as the typical life expectancy *after* an animal has survived its first year.

Unfortunately, we are unable to share all of the individual studbook databases from which our MLE estimates are derived. For users interested in more detailed survival data from studbooks (e.g., age-specific survival rates), please contact the Studbook Keeper(s) and Program Coordinator(s) for the species of interest (an updated listing can be found on the AZA Animal Programs Database https://www.aza.org/about-animal-programs-database). As managers of the Animal Program and the studbook data, they determine the terms and conditions under which to share data or collaborate with researchers. You may also contact us or AZA (conservation@AZA.org) for more information or assistance with inquiries.

## Additional information

**How to cite this article**: Che-Castaldo, J. P. *et al*. Sex-specific median life expectancies from *ex situ* populations for 330 animal species. *Sci. Data*. 6:190019 https://doi.org/10.1038/sdata.2019.19 (2019).

**Publisher’s note**: Springer Nature remains neutral with regard to jurisdictional claims in published maps and institutional affiliations.

## Supplementary Material



## Figures and Tables

**Table 1:** Summary statistics for the median life expectancy estimates (in years) in our dataset by sex (Overall includes male, female, and unknown sex individuals) and by taxonomic group.

Taxon	Overall		Male		Female	
	N	Mean MLE ± SD	N	Mean MLE ± SD	N	Mean MLE ± SD
Amphibia	6	5.37 ± 1.43	6	5.53 ± 1.37	6	5.18 ± 1.53
Arachnida	0		0		1	17.20
Aves	124	12.20 ± 7.34	100	11.39 ± 5.92	107	10.74 ± 6.20
Chondrichthyes	2	16.40 ± 14.57	1	5.90	1	6.50
Mammalia	175	14.71 ± 7.49	139	13.42 ± 6.99	141	14.35 ± 7.54
Reptilia	21	11.63 ± 7.01	14	11.05 ± 7.23	17	10.00 ± 5.34
